# Trends in antibiotic use before and during the coronavirus disease 2019 (COVID-19) pandemic across an integrated health system with different antimicrobial stewardship program models trends in antibiotic use by ASP model

**DOI:** 10.1017/ash.2022.39

**Published:** 2022-04-08

**Authors:** Jennifer M. Peterson, Kristina White, Emily Muehling, Steven C. Ebert, Lisa Lambi, Corey Thieman, Nathan Peterson, Kia Deuel, Amanda M. Bushman, Sudhir Kumar, Leyla A. Best, Rossana Rosa

**Affiliations:** 1 Des Moines University, Des Moines, Iowa; 2 Department of Pharmacy, UnityPoint Health–Peoria, Peoria, Illinois; 3 Department of Pharmacy, System Clinical Services, UnityPoint Health–Des Moines, Des Moines, Iowa; 4 Department of Pharmacy, UnityPoint Health–Meriter, Madison, Wisconsin; 5 Department of Pharmacy, UnityPoint Health–Cedar Rapids, Cedar Rapids, Iowa; 6 Department of Pharmacy, UnityPoint Health–Sioux City, Sioux City, Iowa; 7 Department of Pharmacy, UnityPoint Health–Quad Cities, Bettendorf, Iowa; 8 Department of Infection Prevention, UnityPoint Health–Fort Dodge, Fort Dodge, Iowa; 9 Department of Pharmacy, UnityPoint Health–Des Moines, Des Moines, Iowa; 10 Infectious Diseases Service, UnityPoint Health–Des Moines, Des Moines, Iowa; 11 Department of Internal Medicine, University of Iowa–Des Moines Campus, Des Moines, Iowa

## Abstract

Changes in antimicrobial use during the pandemic in relation to long-term trends in utilization among different antimicrobial stewardship program models have not been fully characterized. We analyzed data from an integrated health system using joinpoint regression and found temporal fluctuations in prescribing as well as continuation of existing trends.

It has been postulated that the coronavirus disease 2019 (COVID-19) pandemic has disrupted the functions of antimicrobial stewardship programs (ASPs), which are considered one of the key tools for mitigating the evolution of multidrug-resistant organisms (MDROs).^1,2^ This concern has arisen since members of the ASPs have been diverted in large part to develop institutional guidance and policies for the management of COVID-19.^3^ Moreover, increases in empiric antibiotic prescribing during the COVID-19 pandemic have been described.^4^ However, the extent to which long-term trends in antimicrobial use across different antimicrobial stewardship program (ASP) models have changed throughout the COVID-19 pandemic is less well understood. We have described temporal fluctuations and longitudinal trends in antimicrobial prescribing of key agents across an integrated healthcare system with different ASP models.

## Methods

This study was conducted at 12 hospitals that are part of an integrated health system in Iowa. Data for antibiotic days of therapy (DOT) and days present were extracted from a centralized database. Only medical–surgical and intensive care units (ICUs) were included. The antibiotics most frequently prescribed at our facilities were selected for analysis: meropenem, piperacillin–tazobactam, cefepime, ceftriaxone, vancomycin, azithromycin, doxycycline, and levofloxacin. None of the antibiotics included are subject to preauthorization. We collected data from January 1, 2019, to February 28, 2021, which encompasses a period prior to the widespread availability of vaccines and preceding the circulation of the δ (delta) and δ (omicron) variants. The prepandemic period was defined as January 1, 2019, to February 29, 2020, and the pandemic period was defined as March 1, 2020, to February 28, 2021. During the first year of the pandemic, the state of Iowa experienced 2 significant increases in community spread with commensurate increase in hospitalizations for COVID-19. The first peak occurred in early May 2020 (peak number of patients hospitalized, 417) and the second peak occurred in mid-November 2020 (peak number of patients hospitalized, 1,510).^5^

### Antimicrobial stewardship program models

The daily antimicrobial stewardship activities and the composition of the staff performing them varied by site. Stewardship activities remained unchanged throughout the study period. The characteristics of the hospitals supported by each ASP model, as well as the ASP members and workflow are described in Table 1.

**Table 1.** Hospital and Antimicrobial Stewardship Program (ASP) Model Characteristics

Model	Hospital characteristics	ASP members	ASP workflow
Model A	370 beds, level I trauma center, intensive care, oncology, kidney transplant units 224 beds, ICU 95 beds hospital, no ICU All urban facilities	ID physician on site ID pharmacist ID nurse	The ASP meets from Monday to Friday to review positive cultures from sterile sites and the use of broad-spectrum antibiotics. Pertinent therapeutic recommendations are communicated to the primary team via message on the EMR or directly by phone call, depending on the urgency of the recommendation.
Model B	40 beds, ICU Semirural	ID physician off site Clinical pharmacist Infection control nurse	The ASP meets virtually through videoconference on Mondays, Wednesdays, and Fridays to review positive culture from sterile sites and the use of broad-spectrum antibiotics. Recommendations are communicated to the primary team via progress notes in the EMR.
Model C	229 beds, ICU, oncology unit 371 beds, level III trauma center, ICU 106 beds, ICU 191 beds, level III trauma center, ICU 453 beds distributed in a 4-medical centers complex, level II trauma center, oncology unit	Clinical pharmacists (4 regions) ID pharmacist (1 region)	The clinical pharmacist performs a daily review of positive cultures and antimicrobial lists utilizing alerts set through an electronic clinical decision-support program. Recommendations are communicated to the primary team by the clinical pharmacists via text message or phone call.

Note. ICU, intensive care unit; ID, infectious disease.

### Statistical analysis

We examined the trends in antibiotic DOT per 1,000 days present among the 3 different ASP models. We used joinpoint regression to determine the number of joinpoints, the monthly percentage changes (MPCs), and the average monthly percent changes (AMPCs).^6^ The joinpoints are points at which the trend changes. The MPC characterizes changes in trends in antibiotic DOT rates occurring at any point during the entire observation period. The AMPC summarizes the trend over prespecified fixed intervals, which in our study were the prepandemic period (January 2019–February 2020) and the pandemic period (March 2020–February 2021). Models were fit to log-transformed antibiotic DOT rates, and permutation analysis was used to select the best-fit model. An autocorrelated error structure was selected to account for autoregression in prescribing rates over time. Analyses were conducted on the Joinpoint Regression Program version 4.7 software (National Cancer Institute, Bethesda, MD).

## Results

### Facilities using antimicrobial stewardship model A

In these facilities, estimates of the MPC for the entire observation showed significant changes in trend of use of piperacillin–tazobactam, ceftriaxone, azithromycin and doxycycline (Table 2, Supplementary Fig. 1B, 1D, 1F, and 1G). During the prepandemic period, AMPC estimates showed monthly increases in the use of ceftriaxone, vancomycin, azithromycin, and a decrease in levofloxacin use (Supplementary Table 1). During the pandemic period, the AMPC showed monthly increases in the use of cefepime, piperacillin–tazobactam, and vancomycin. The AMPC of vancomycin was the same for both the prepandemic and pandemic periods (+0.4%; 95% CI, 0.1–07; *P* = .04), reflecting a longstanding trend.

**Table 2.** Monthly Percent Change in Antibiotic Days of Therapy Per 1,000 Days Present According to Antimicrobial Stewardship Program (ASP) Model, January 1, 2019, to February 28, 2021

Variable	Monthly Change, % (95% CI)	*P* Value
**ASP model A**		
Meropenem		
January 2019–February 2021	−0.2 (−1.3 to 1.1)	.70
Piperacillin–Tazobactam		
January 2019–July 2019	5.1 (1.8 to 8.6)	.04
July 2019–January 2020	−3.4 (−7.4 to 8.6)	.10
January 2020–February 2021	1.3 (0.3 to 2.6)	.01
Cefepime		
January 2020–February 2021	3.2 (2.4 to 3.9)	<.01
Ceftriaxone		
January 2019–November 2019	1.6 (0.5 to 2.8)	.01
November 2019–April 2020	7.3 (2.4 to 12.7)	.01
April 2020–July 2020	−5.2 (−24.2 to 17.4)	.60
July 2020–February 2021	1.5 (−0.3 to 4.6)	.10
Vancomycin		
January 2019–February 2021	0.4 (0.01 to 0.7)	.04
Azithromycin		
January 2019–August 2019	−1.9 (−5 to 1.3)	.20
August 2019–February 2020	9.6 (4.3 to 15)	.03
February 2020–May 2020	−15.7 (−45.7 to 31.1)	.40
May 2020–August 2020	13.1 (−20.9 to 61.8)	.50
August 2020–February 2021	−0.8 (−4.5 to 3.2)	.70
Doxycycline		
January 2019–January 2020	−0.6 (−4.3 to 3.3)	.80
January 2020–April 2020	57.9 (−31.7 to 264.9)	.30
April 2020–August 2020	−25.1 (−43.8 to −2.6)	.03
August 2020–February 2021	6.3 (−5 to 25.7)	.20
Levofloxacin		
January 2019–June 2020	−3.0 (−3.9 to −2)	<.01
June 2020–February 2021	2 (−1.5 to 5.6)	.30
**ASP model B**		
Meropenem		
January 2019–February 2021	2.3 (0.8 to 3.8)	<.01
Piperacillin−tazobactam		
January 2019–February 2021	0.1 (−0.5 to 0.8)	.70
Cefepime		
January 2019–March 2020	7.0 (2.6 to 9)	<.01
March 2020–June 2020	−25.0 (−74.4 to 120.1)	.60
June 2020–February 2021	7.0 (−1.0 to 9.0)	.10
Ceftriaxone		
January 2019–February 2021	1.0 (0.2 to 1.7)	.02
Vancomycin		
January 2019–February 2021	2.4 (1.0 to 3.5)	<.01
Azithromycin		
January 2019–December 2020	2.2 (0.7 to 3.7)	<.01
December 2020–February 2021	−36.5 (−84.2 to 154.3)	.50
Doxycycline		
January 2019–November 2019	−8.1 (−17 to 1.8)	.10
November 2019–April 2020	29.2 (−16.8 to 100.7)	.20
April 2020–February 2021	−10.7 (−17.9 to −2.9)	.10
Levofloxacin		
January 2019–February 2021	−2.4 (−3.3 to −1.5)	<.01
**ASP model C**		
Meropenem		
January 2019–January 2020	2.5 (0.6 to 4.5)	.01
January 2020–April 2020	19.1 (−23.1 to 84.3)	.40
April 2020–September 2020	−7.1 (−16.2 to 3)	.10
September 2020–December 2020	13.9 (−23.4 to 69.2)	.50
December 2020–February 2021	−16.6 (−47.2 to 31.6)	.40
Piperacillin–tazobactam		
January 2019–February 2021	0.6 (0.2 to 1.0)	<.01
Cefepime		
January 2019–December 2019	−1.7 (−2.9 to −0.4)	.01
December 2019–March 2020	12.2 (−13.7 to 43.2)	.40
March 2020–July 2020	−7.8 (−18.3 to 4)	.20
July 2020–December 2020	8.3 (2.1 to 15)	<.01
December 2020–February 2021	−16.6 (−36.4 to 9.3)	.20
Ceftriaxone		
January 2019–February 2021	0.9 (0.4 to 1.3)	<.01
Vancomycin		
January 2019–February 2021	−0.3 (−0.5 to −0.1)	.05
Azithromycin		
January 2019–July 2019	5.3 (−8.2 to −2.3)	<.01
July 2019–April 2020	7.2 (5.2 to 9.2)	<.01
April 2020–August 2020	−18.2 (−34.2 to 1.6)	.1
August 2020–November 2020	19.7 (10.2 to 29.9)	<.01
November 2020–February 2021	−16.8 (−23.9 to −9.1)	.04
Doxycycline		
January 2019–May 2020	1.6 (0.2 to 3)	.03
May 2020–February 2021	−3.6 (−7 to 0.1)	.03
Levofloxacin		
January 2019–July 2019	−3.6 (−5.6 to −1.6)	<.01
July 2019–January 2020	0.7 (−2.1 to 3.5)	.60
January 2020–October 2020	−3.3 (−4.7 to −1.9)	<.01
October 2020–February 2021	2.6 (−2.5 to 7.9)	.30

### Facility using antimicrobial stewardship model B

At this site, estimates of the MPC for the entire observation period detected significant changes in the trends in cefepime and azithromycin use (Table 2 and Supplementary Fig. 2C and 2F). For the prepandemic period, AMPC estimates showed monthly increases in the use of meropenem, cefepime, ceftriaxone, vancomycin, and azithromycin, and a decrease in levofloxacin use (Supplementary Table 1). For the pandemic period, AMPCs showed monthly increases in the use of meropenem, ceftriaxone and vancomycin, and a decrease in levofloxacin use (Supplementary Table 1). The AMPCs for meropenem (+2.3; 95% CI, 0.8–3.8; *P* < .01), ceftriaxone (+1.0; 95% CI, 0.2–1.7; *P* = .02), vancomycin (+2.4; 95% CI, 1.0–3.5; *P* < .01), and levofloxacin (−2.4; 95% CI, −3.3 to −1.5; *P* < .01) remained the same for the prepandemic and pandemic periods, reflecting longstanding trends.

### Facilities using antimicrobial stewardship model C

In these facilities, the MPC estimates for the entire observation period indicated multiple significant fluctuations in the use of meropenem, cefepime, azithromycin, doxycycline, and levofloxacin (Table 2 and Supplementary Figs 3A, 3C, 3F, 3G and 3H). For the prepandemic period, the AMPCs showed monthly increases in the use of meropenem, piperacillin–tazobactam, ceftriaxone, and doxycycline, and a decreases in the use of vancomycin and levofloxacin (Supplementary Table 1). For the pandemic period, the AMPC showed monthly increases in the use of piperacillin–tazobactam and ceftriaxone and a decrease in vancomycin use (Table 2). The AMPCs of piperacillin–tazobactam (+0.6; 95% CI, 0.2–1; *P* < .01), ceftriaxone (+0.9; 95% CI, 0.4–1.3; *P* < .01), and vancomycin (−0.3; 95% CI, −0.5 to −0.1; *P* = .05) remained the same for both the prepandemic and pandemic periods, reflecting the longstanding trend.

## Discussion

Across hospitals using different ASP models, we identified multiple fluctuations in the rates of antibiotic use throughout the study period. In most cases, the average monthly percent changes reflected trends that preceded the COVID-19 pandemic.

Up to 75% of patients with COVID-19 are prescribed antibiotics, and rates of prescribing have decreased throughout the pandemic.^4^ Our assessment of longitudinal trends in prescribing revealed fluctuations that, in most instances, did not reach a statistically significant deviation from the existing trend. There is a need for development of ASPs in settings with both limited access to the expertise of infectious diseases specialists, particularly during the COVID-19 pandemic, as well as a lack of reports of stewardship practices.^7^ Our study contributes to the literature on this topic by describing 3 ASP models in urban and semirural areas and by describing trends of use of key antibiotic agents under different stewardship practices.

The limitations of our study include use of registry-type data obtained from a centralized database in which accuracy depends on appropriate capture of data in the medication administration record. This aspect is mitigated by previous reviews on the accuracy of this data performed by clinical pharmacists. Also, we did not have data for the precise indication of antibiotic prescribing, which precluded us from evaluating changes in trends used specifically for respiratory infection. Furthermore, we did not assess the role of individual tools for mitigating antimicrobial use such as procalcitonin trends.^8^

In conclusion, across 3 different ASP models, the core stewardship activities were maintained during the COVID-19 pandemic. Changes in antibiotic use were limited to temporal fluctuations and, for most agents, longstanding trends continued. Continued support and development of ASPs in accordance with local resources is crucial to their success.

## Supplementary material

For supplementary material accompanying this paper visit https://doi.org/10.1017/ash.2022.39. click here to view supplementary material
