# Supplementary material for: Trends in antibiotic use before and during the coronavirus disease 2019 (COVID-19) pandemic across an integrated health system with different antimicrobial stewardship program models trends in antibiotic use by ASP model
Source: Antimicrob Steward Healthc Epidemiol. 2022 Apr 8;2(1):e55. doi: 10.1017/ash.2022.39 (PMC9726599; doi:10.1017/ash.2022.39)

Supplemental Table 1. Average monthly percent change in antibiotic days of therapy per 1000 days present according to Antimicrobial Stewardship Program (ASP) model

|  | Average monthly percent change  Entire period  January 2019-  February 2021 | *P* | Average monthly percent change  Pre-pandemic period  January 2019-  February 2020 | *P* | Average monthly percent change  Pandemic period  March 2020-  February 2021 | ­*P* |
| --- | --- | --- | --- | --- | --- | --- |
| ***ASP Model A*** |  | | | | | |
| Meropenem | -0.2 (-1.3-1.1) | 0.70 | -0.2 (-1.3-1.1) | 0.70 | -0.2 (-1.3-1.1) | 0.70 |
| Piperacillin-Tazobactam | 1.1(-0.2-2.4) | 0.10 | 0.8(-1.5-3.1) | 0.50 | 1.4(0.3-2.6) | 0.01 |
| Cefepime | 5 (0.9-9.3) | 0.01 | 5.2(-1.9-12.7) | 0.20 | 4.8(1.7-7.9) | <0.01 |
| Ceftriaxone | 2(0.8-4.8) | 0.20 | 2.9(1.6-4.3) | <0.01 | 0.2(-5.7-6.6) | 0.90 |
| Vancomycin | 0.4(0.01-0.7) | 0.04 | 0.4(0.01-0.7) | 0.04 | 0.4(0.01-0.7) | 0.04 |
| Azithromycin | 0.9(-5.3-7.5) | 0.80 | 3.2(0.6-5.9) | 0.03 | -0.2(-11.0-12.0) | 0.99 |
| Doxycycline | 2.0(-8.3-13.5) | 0.70 | 1.2(-0.3-2.8) | 0.10 | -3.3(-9.1-2.8) | 0.30 |
| Levofloxacin | -1.4(-2.6- -0.2) | 0.20 | -3(-3.9- -2) | <0.01 | 0.6(-1.8-3.1) | 0.60 |
| ***ASP Model B*** |  | | | | | |
| Meropenem | 2.3(0.8-3.8) | <0.01 | 2.3(0.8-3.8) | <0.01 | 2.3(0.8-3.8) | <0.01 |
| Piperacillin-Tazobactam | 0.1(-0.5-0.8) | 0.70 | 0.1(-0.5-0.8) | 0.70 | 0.1(-0.5-0.8) | 0.70 |
| Cefepime | 2.5(--9.5-16.1) | 0.70 | 7(3.5-10.6) | <0.01 | -2.9(-26.6-28.5) | 0.80 |
| Ceftriaxone | 1(0.2-1.7) | 0.02 | 1(0.2-1.7) | 0.02 | 1(0.2-1.7) | 0.02 |
| Vancomycin | 2.4 (1.0-3.5) | <0.01 | 2.4 (1.0-3.5) | <0.01 | 2.4 (1.0-3.5) | <0.01 |
| Azithromycin | -1.6(-11.5-9.3 | 0.80 | 2.2(0.7-3.7) | <0.01 | -6.3(-26.2-18.9) | 0.60 |
| Doxycycline | 2.7(-11.6-7) | 0.60 | -0.6(-11.8-12.1) | 0.90 | -3(-14.8-10.4) | 0.60 |
| Levofloxacin | -2.4(-3.3- -1.5) | <0.01 | -2.4(-3.3- -1.5) | <0.01 | -2.4(-3.3- -1.5) | <0.01 |
| ***ASP Model C*** |  | | | | | |
| Meropenem | 1.9(-5.4-9.8) | 0.60 | 3.7(0.1-7.4) | 0.03 | -1.5(-13.9-12.7) | 0.80 |
| Piperacillin-Tazobactam | 0.6(0.2-1) | <0.01 | 0.6(0.2-1) | <0.01 | 0.6(0.2-1) | <0.01 |
| Cefepime | -0.05(-4.4-3.5) | 0.80 | 0.3(-3.3-4.2) | 0.90 | -2.6(-8.6-3.9) | 0.40 |
| Ceftriaxone | 0.9(0.4-1.3) | <0.01 | 0.9(0.4-1.3) | <0.01 | 0.9(0.4-1.3) | <0.01 |
| Vancomycin | -0.3(-0.5- -0.1) | 0.05 | -0.3(-0.5- -0.1) | 0.05 | -0.3(-0.5- -0.1) | 0.05 |
| Azithromycin | -0.6(-3.3-2.3) | 0.70 | 1.2(-0.3-2.8) | 0.10 | -3.3(-9.1-2.8) | 0.30 |
| Doxycycline | -0.3(-1.8-1.2) | 0.70 | 1.6(0.2-3); 0.03 | 0.03 | -2.7(-5.3-0.1) | 0.10 |
| Levofloxacin | -1.5(-2.6- -0.3) | 0.01 | -1.6(-3.1- -0.2) | 0.02 | -1.2(-3-0.7) | 0.2 |


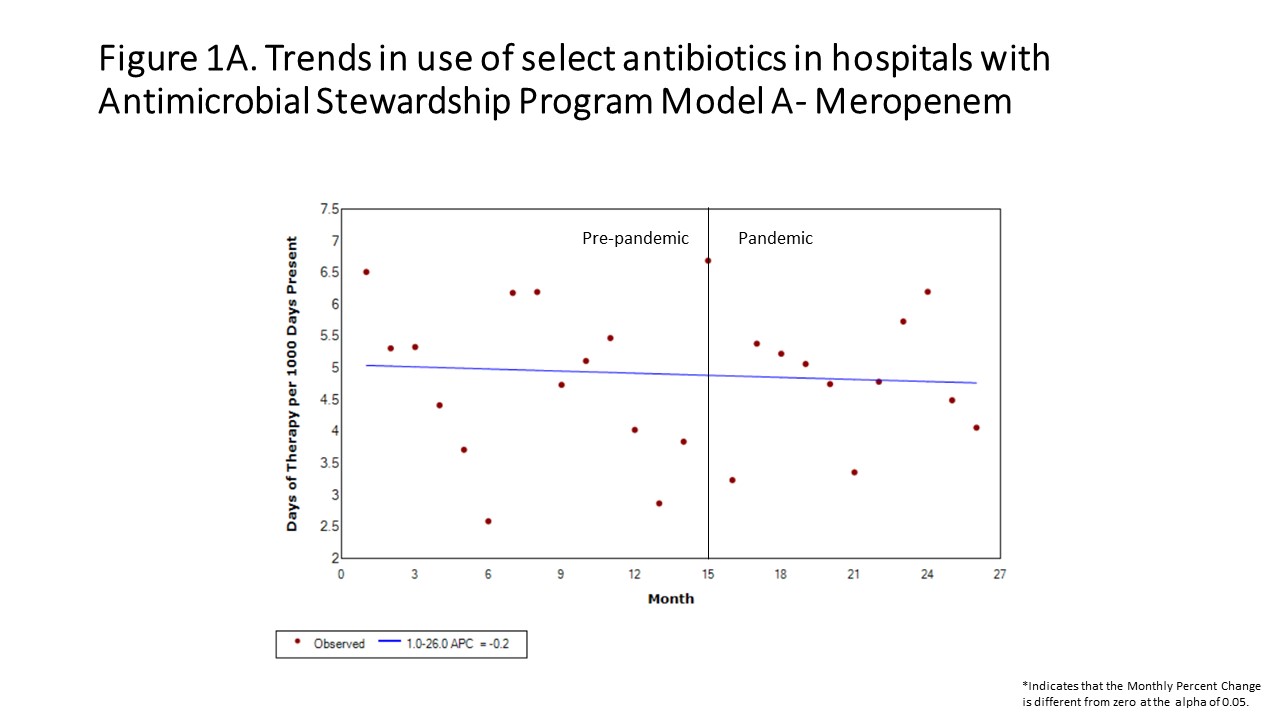


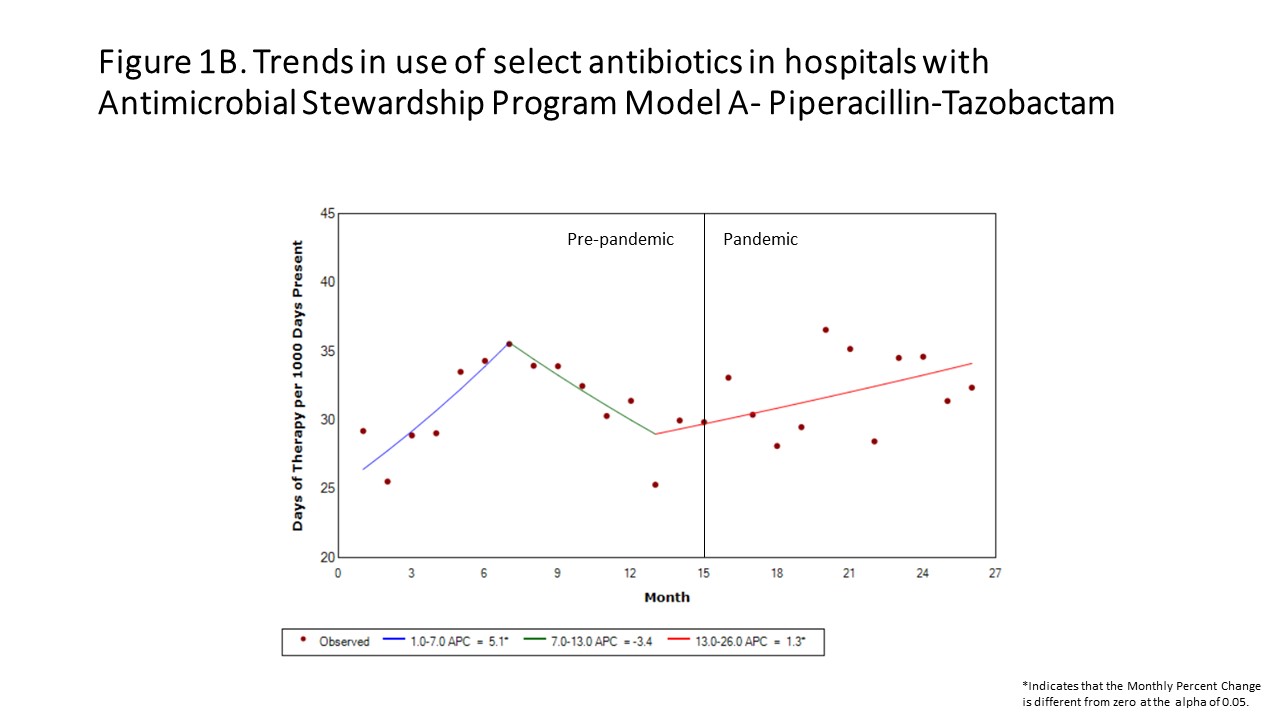


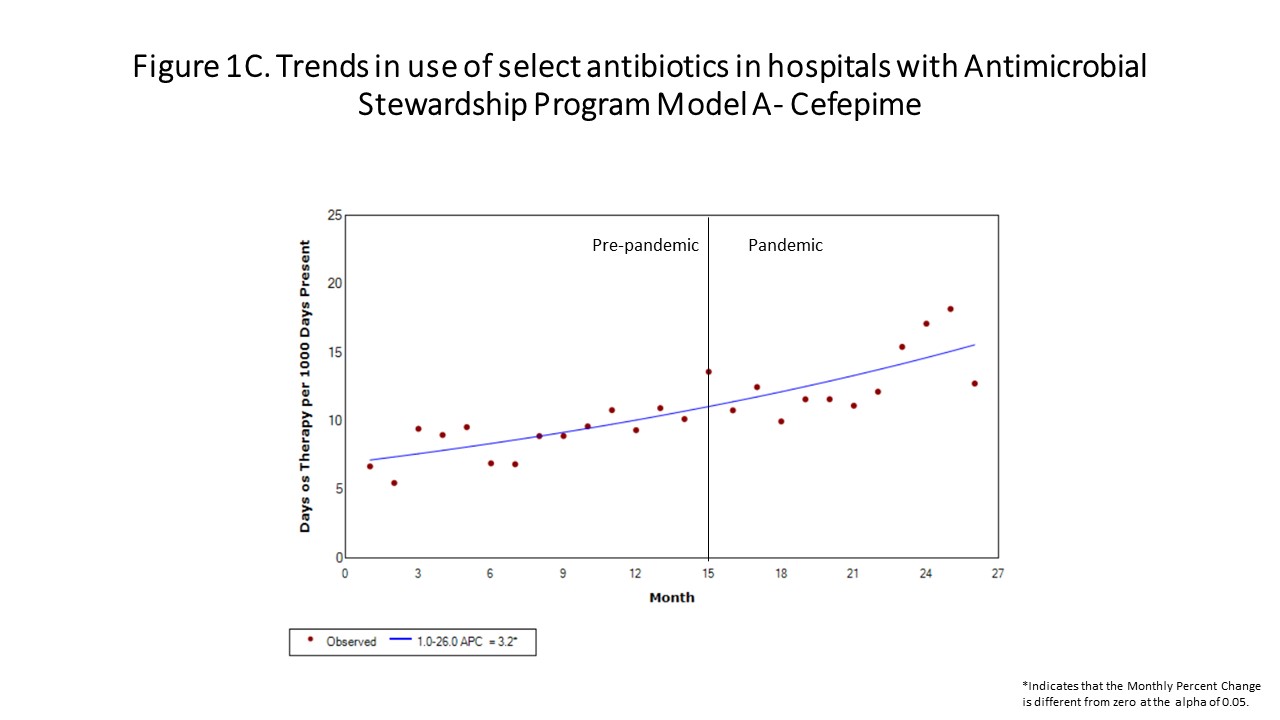


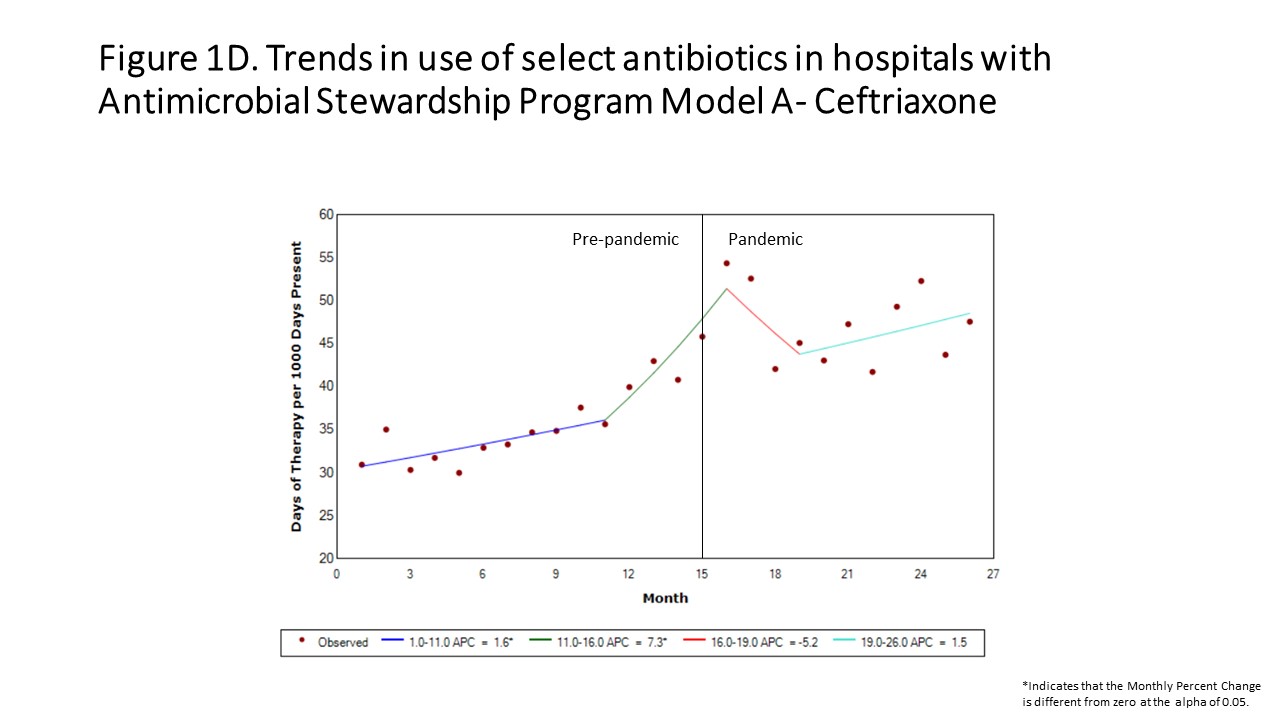


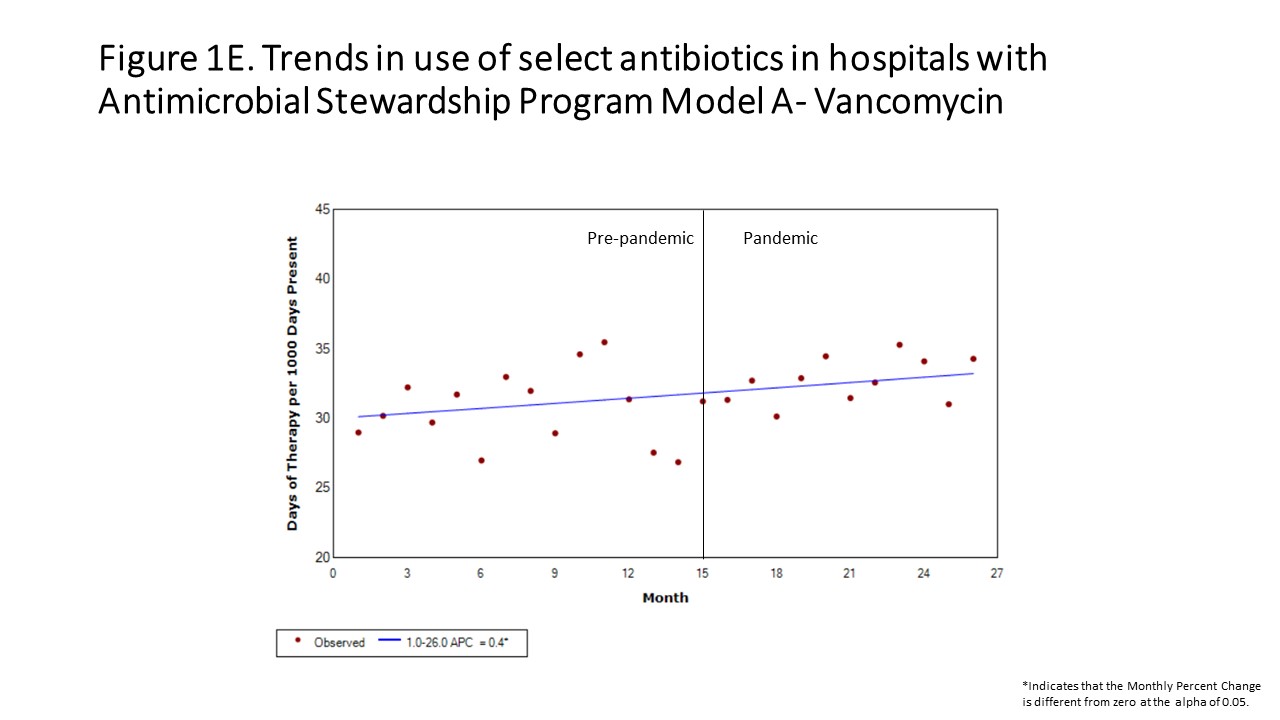


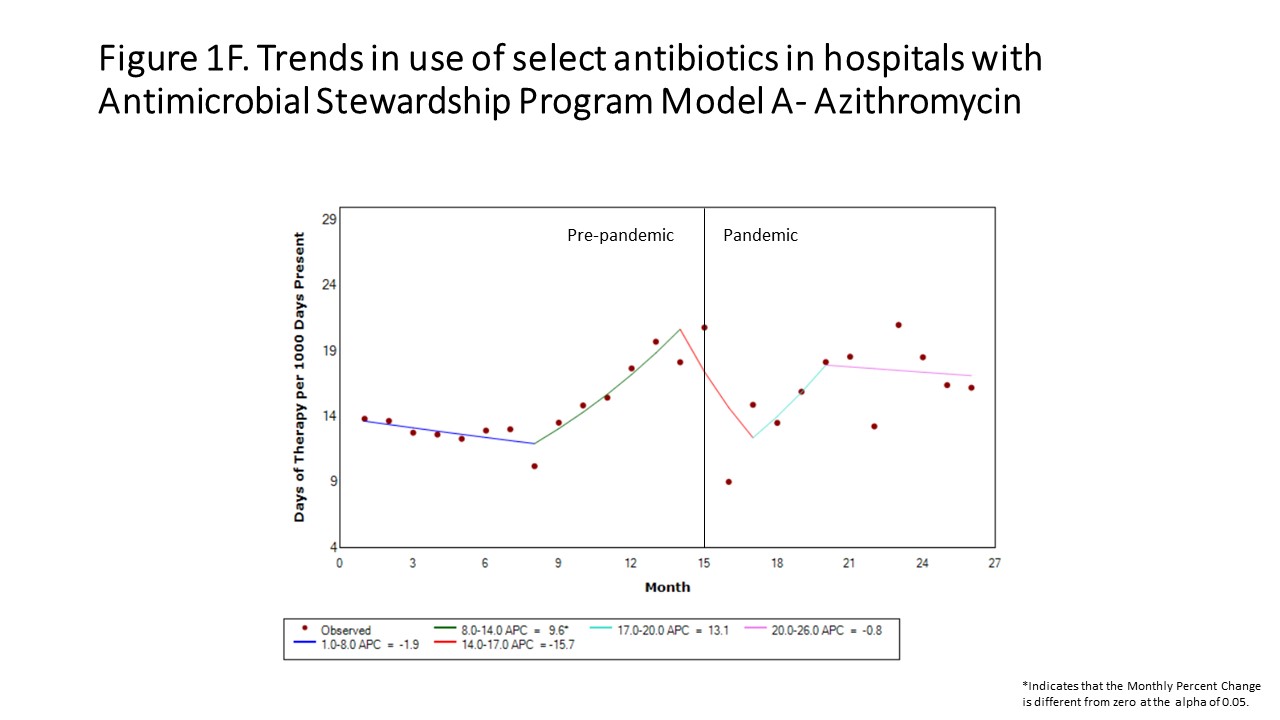


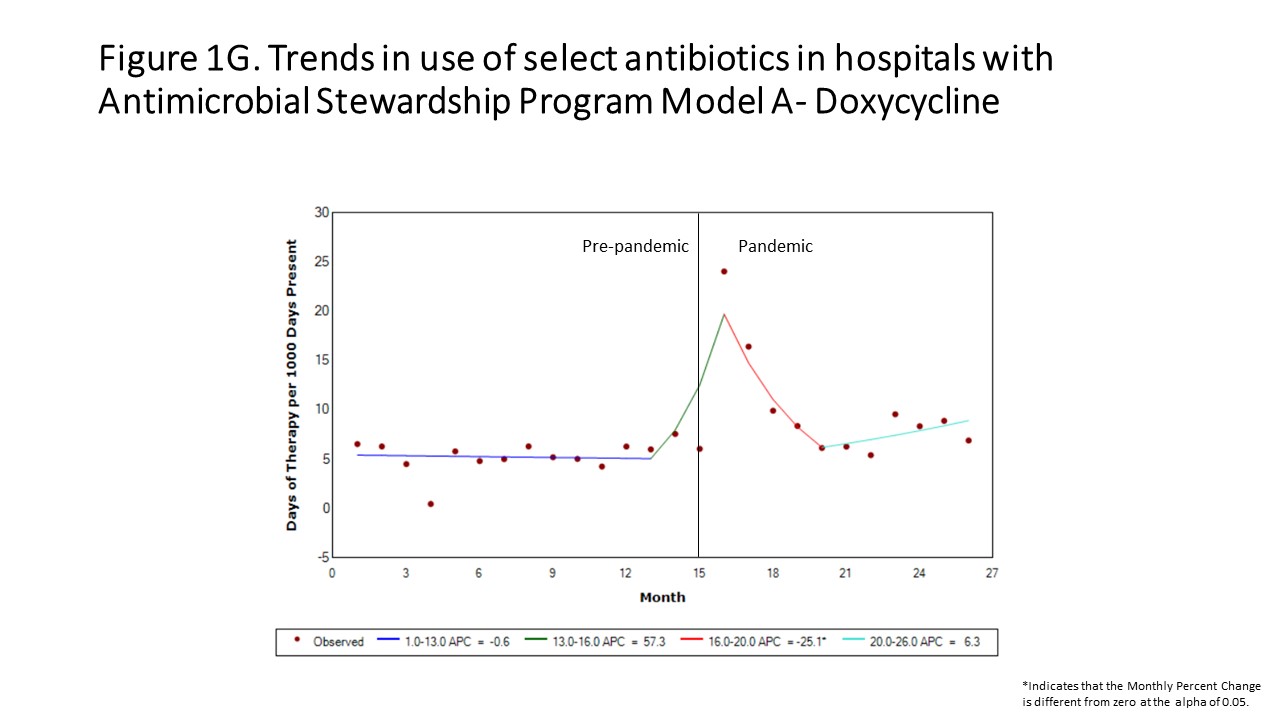


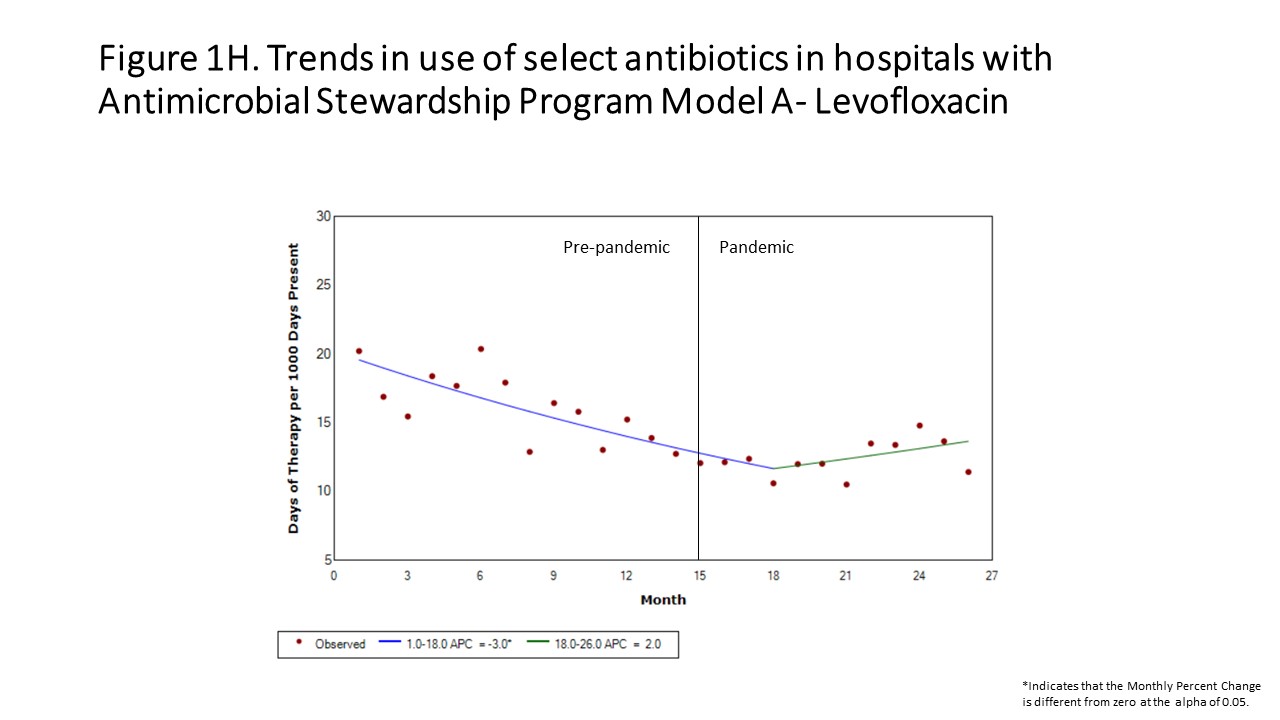


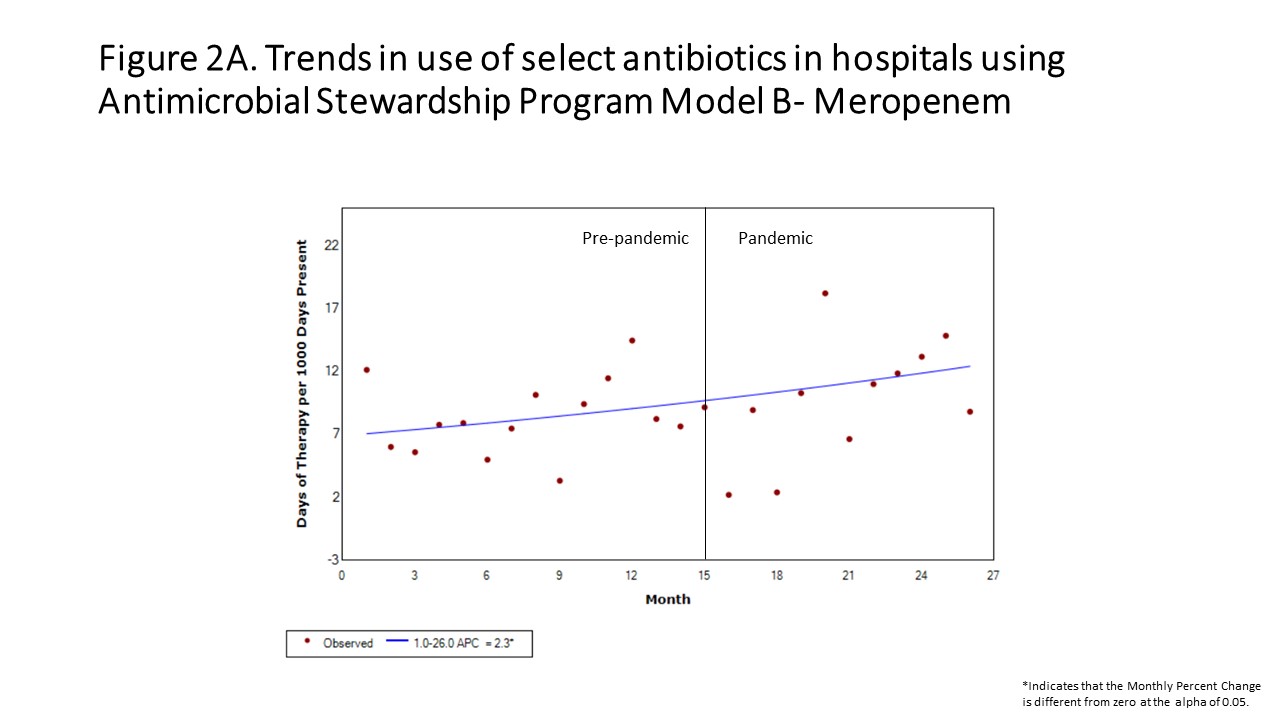


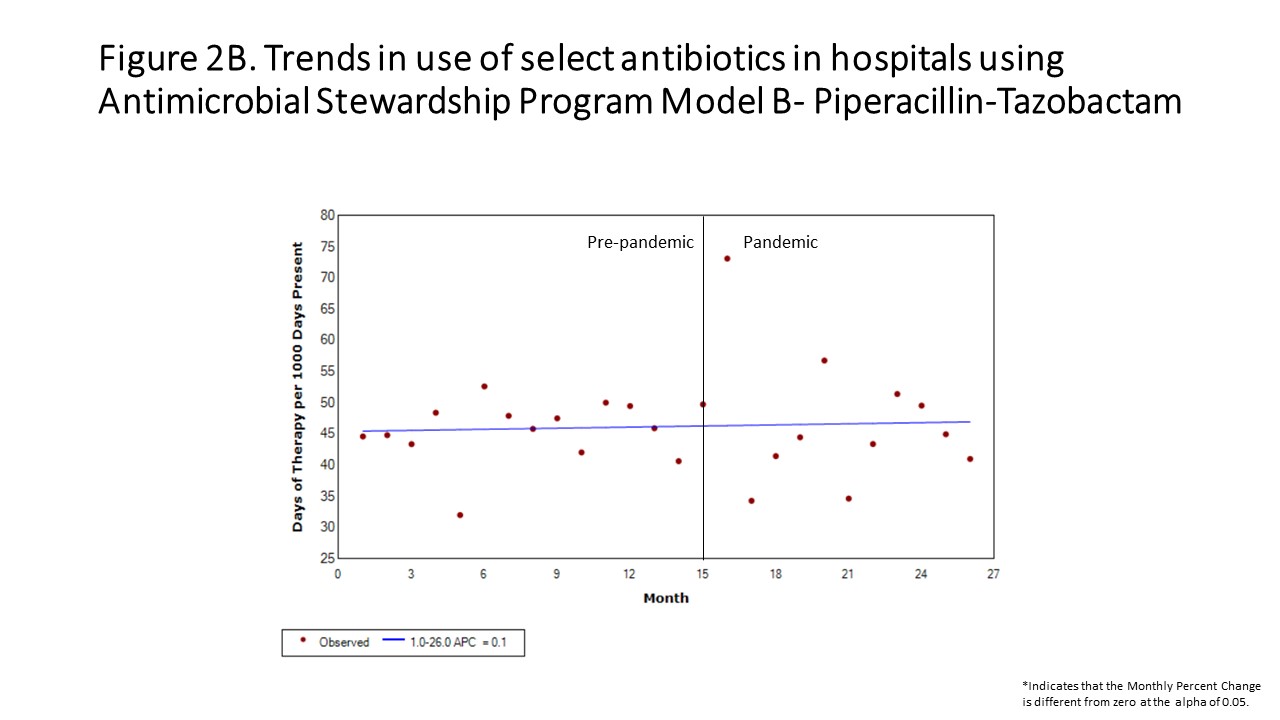


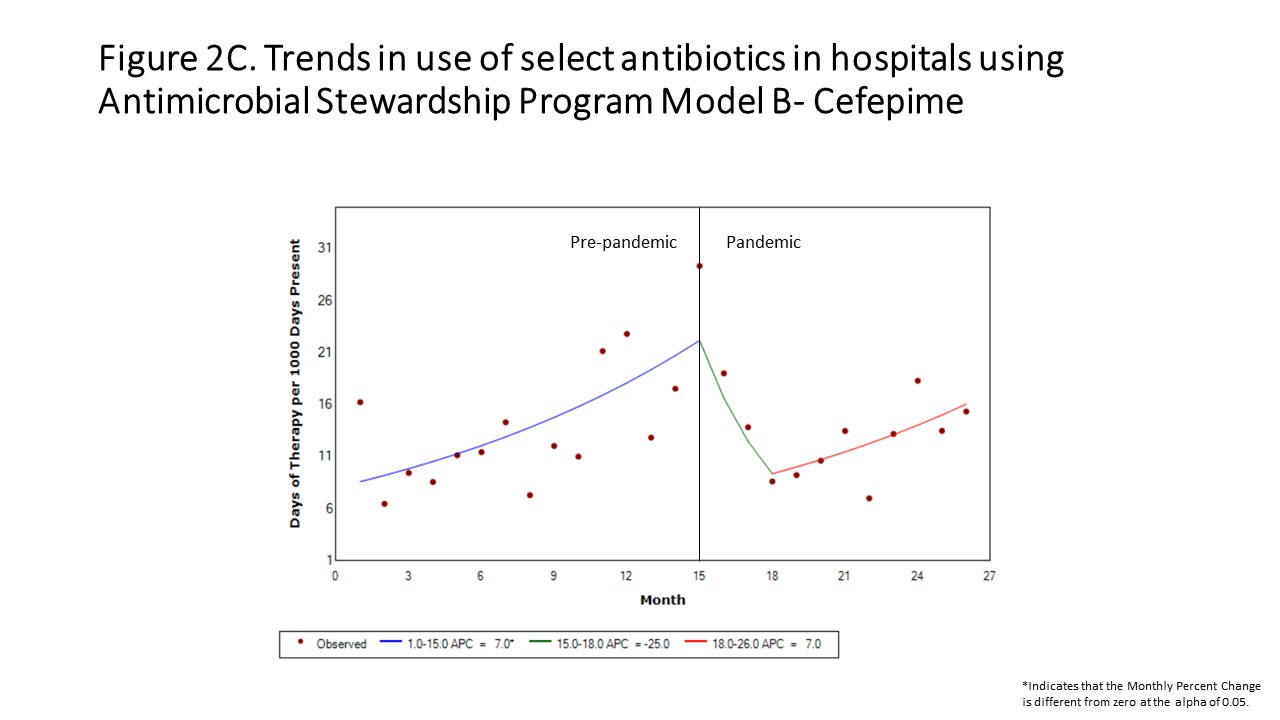


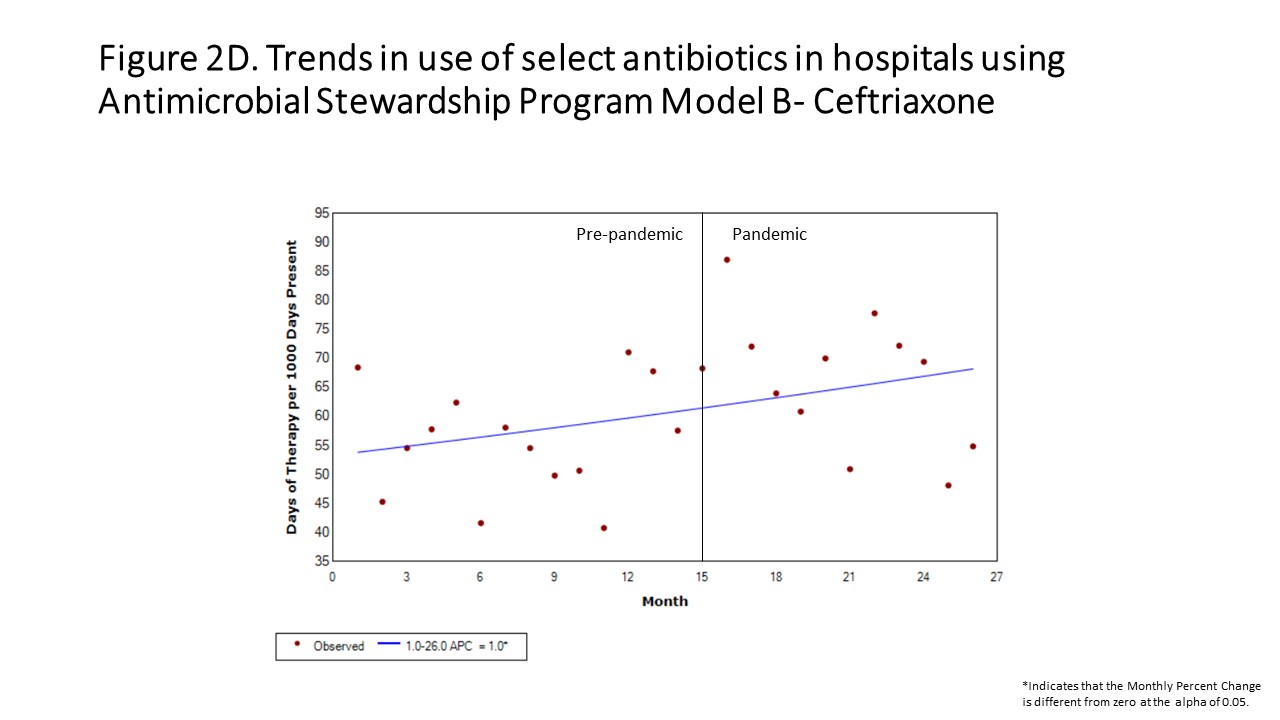


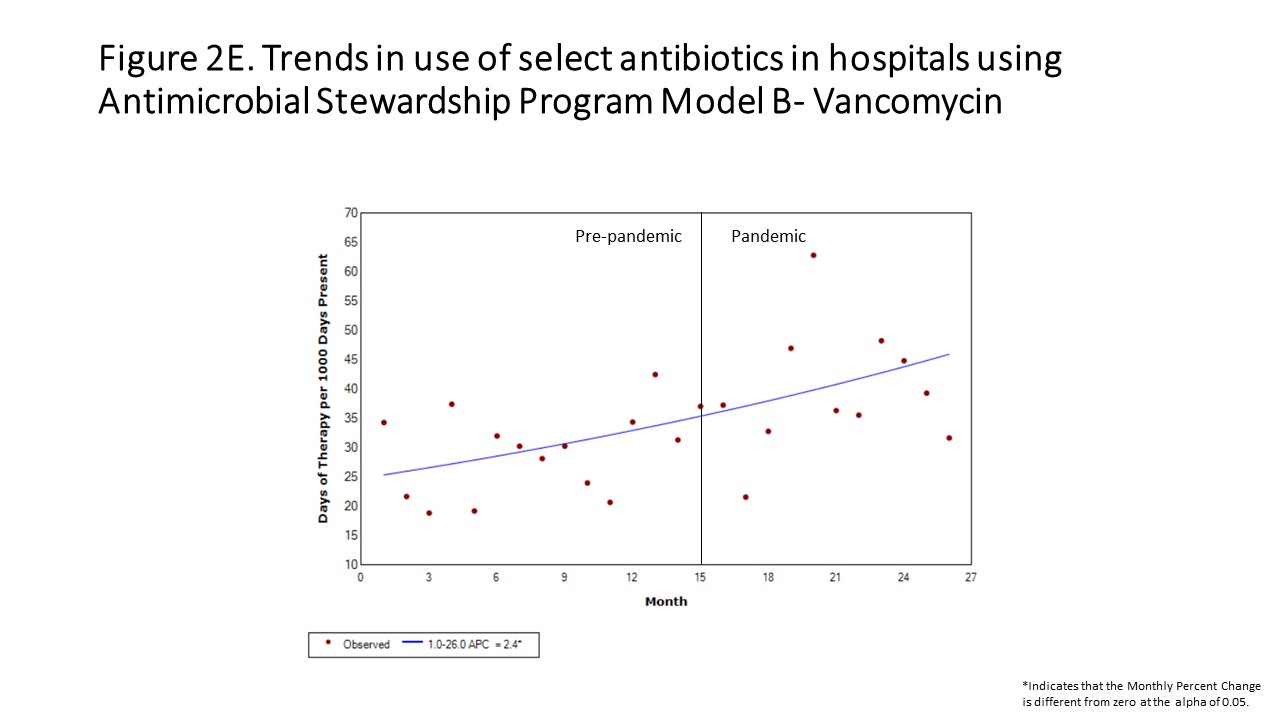


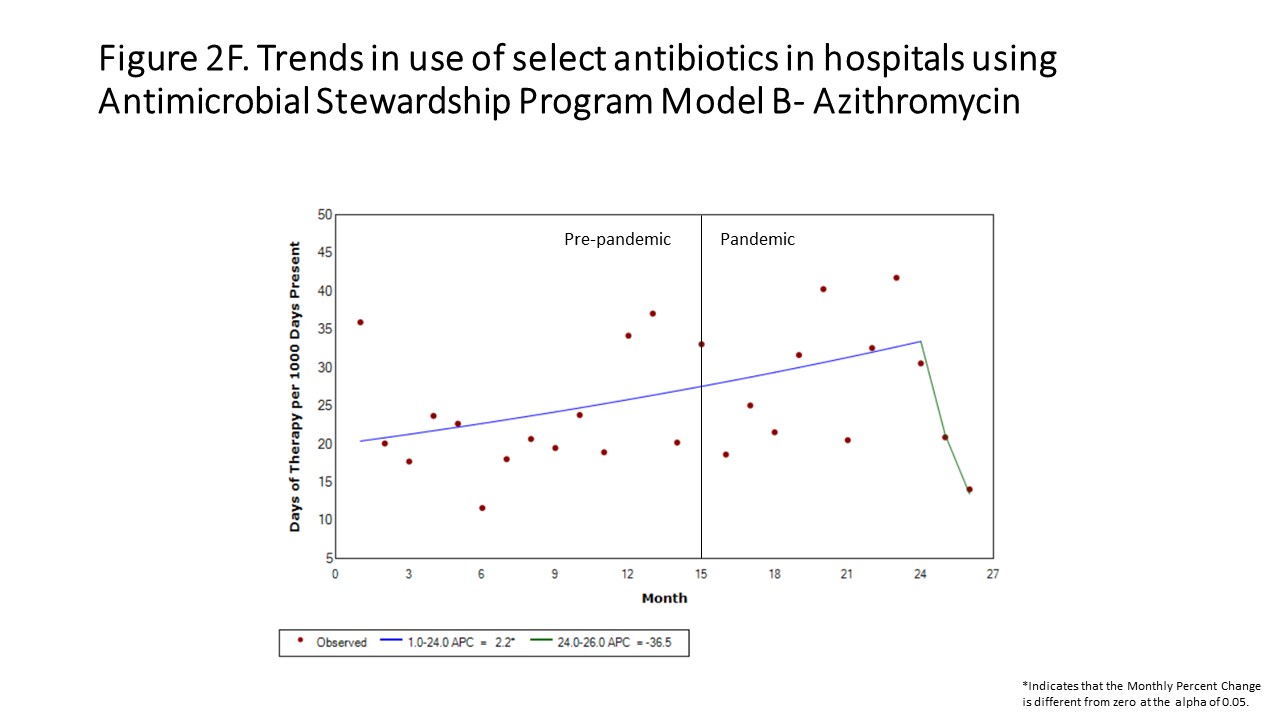


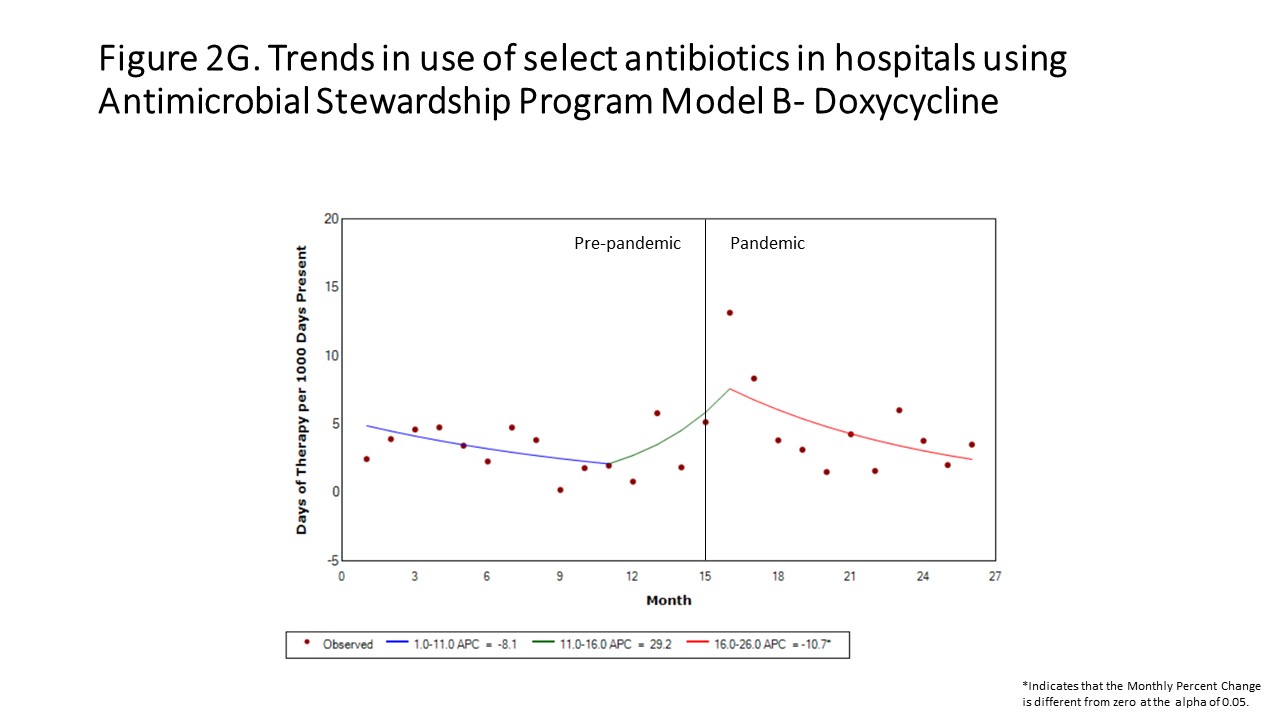


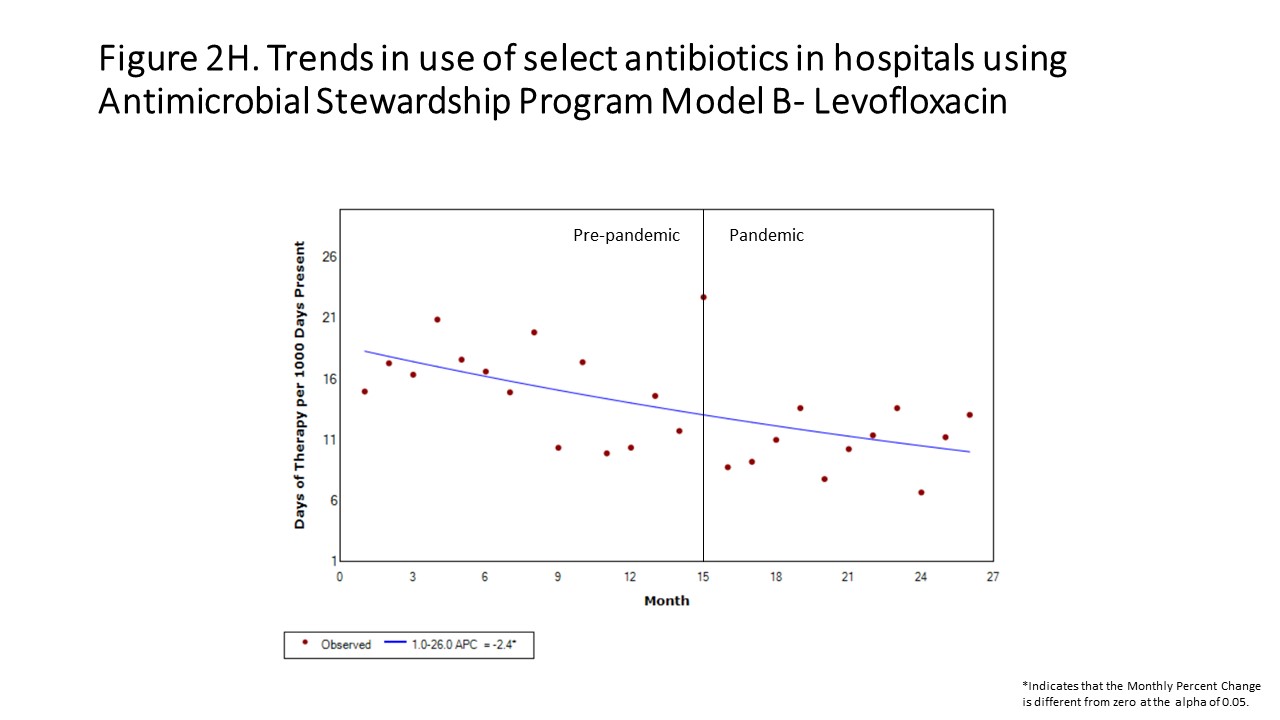


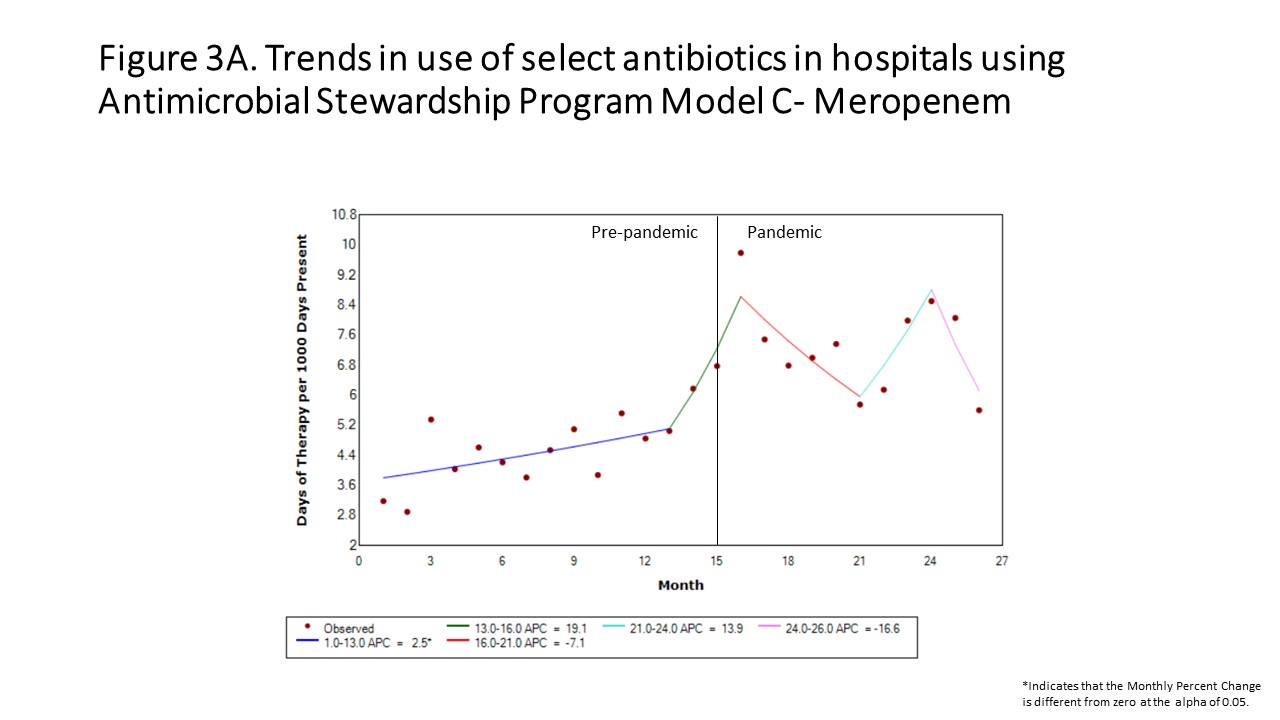


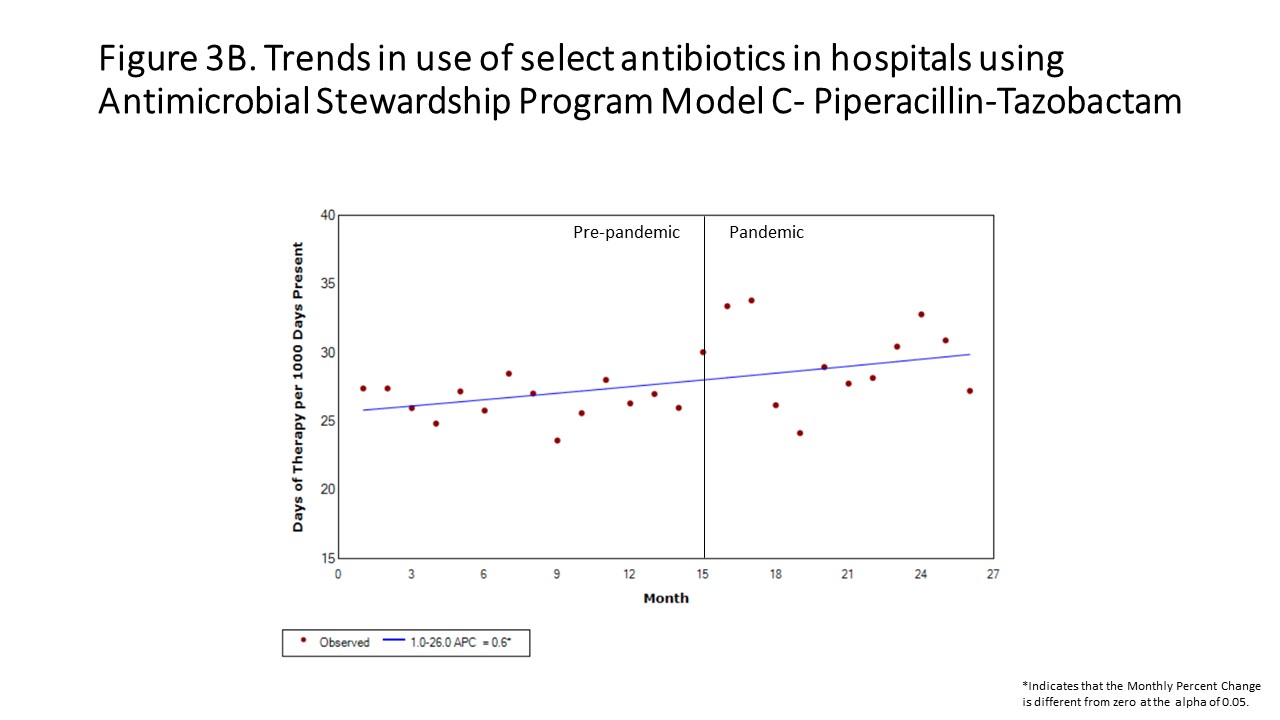


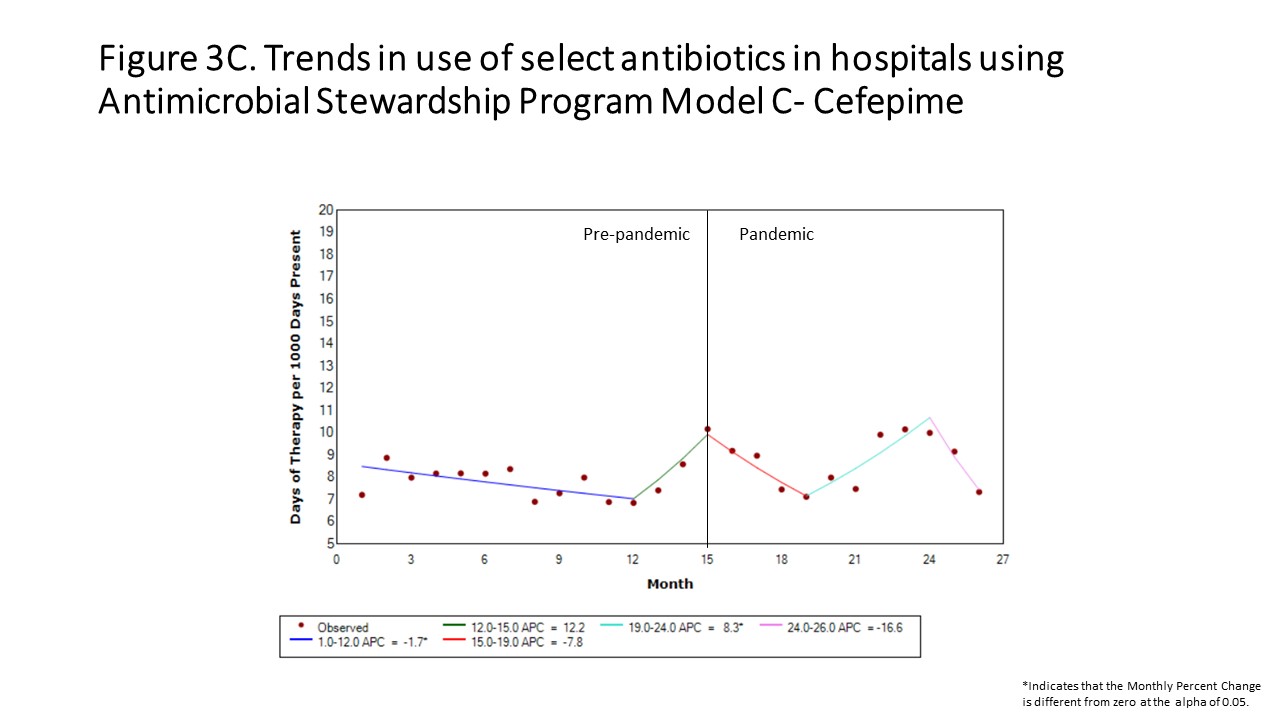


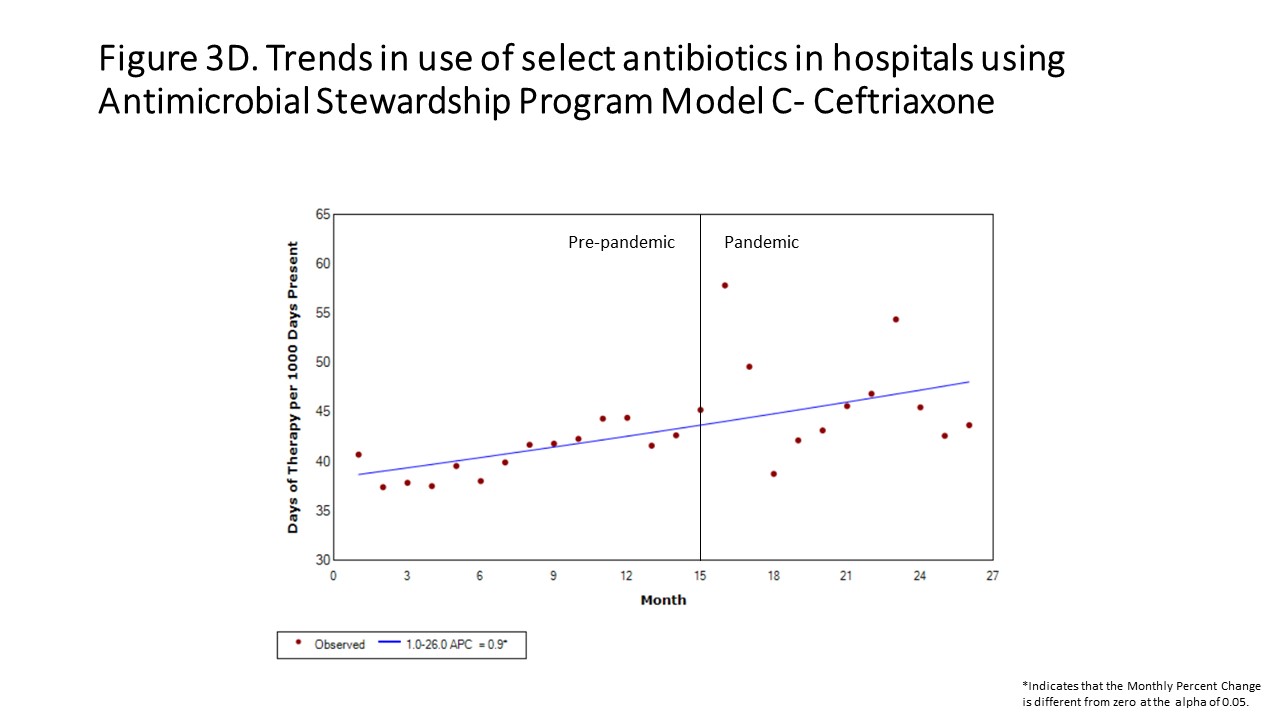


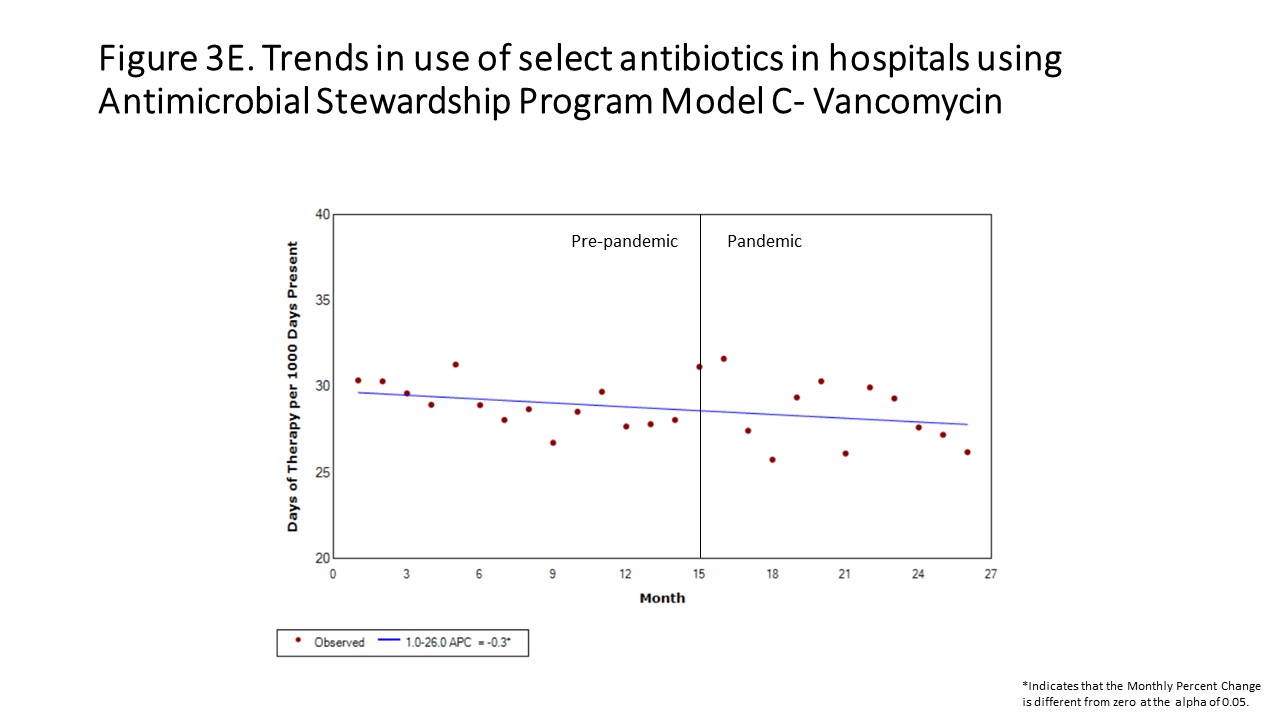


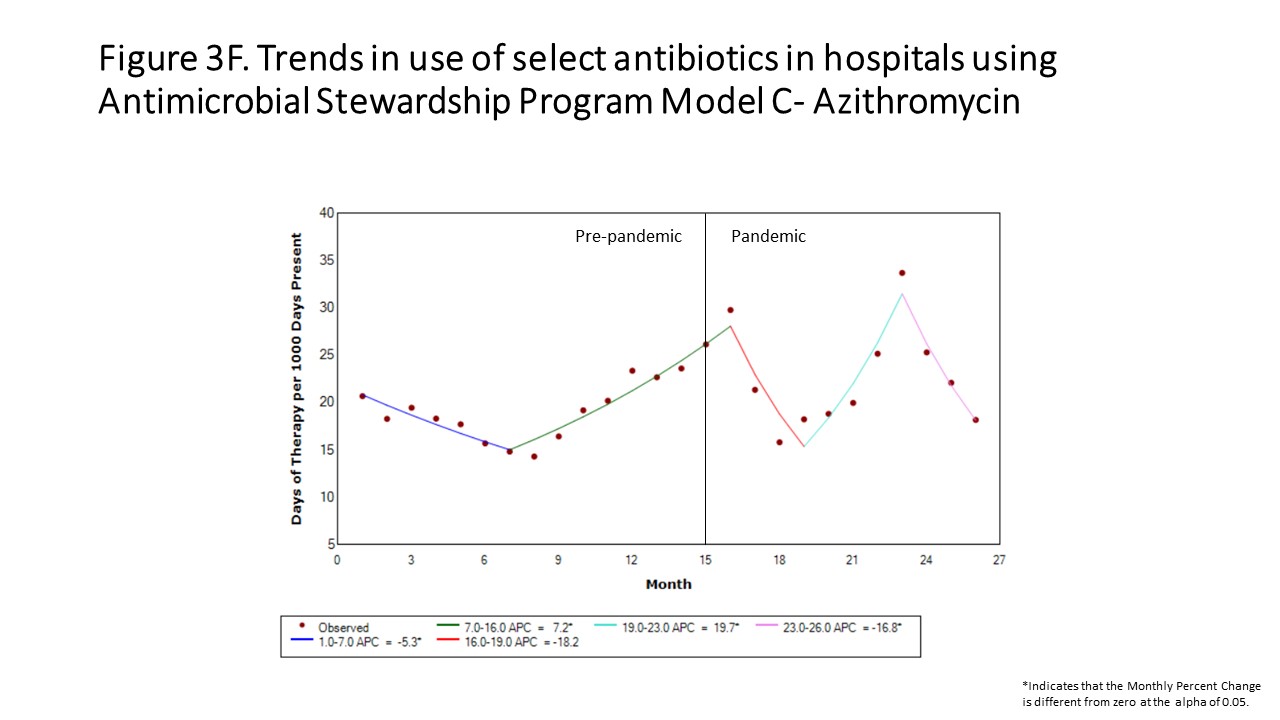


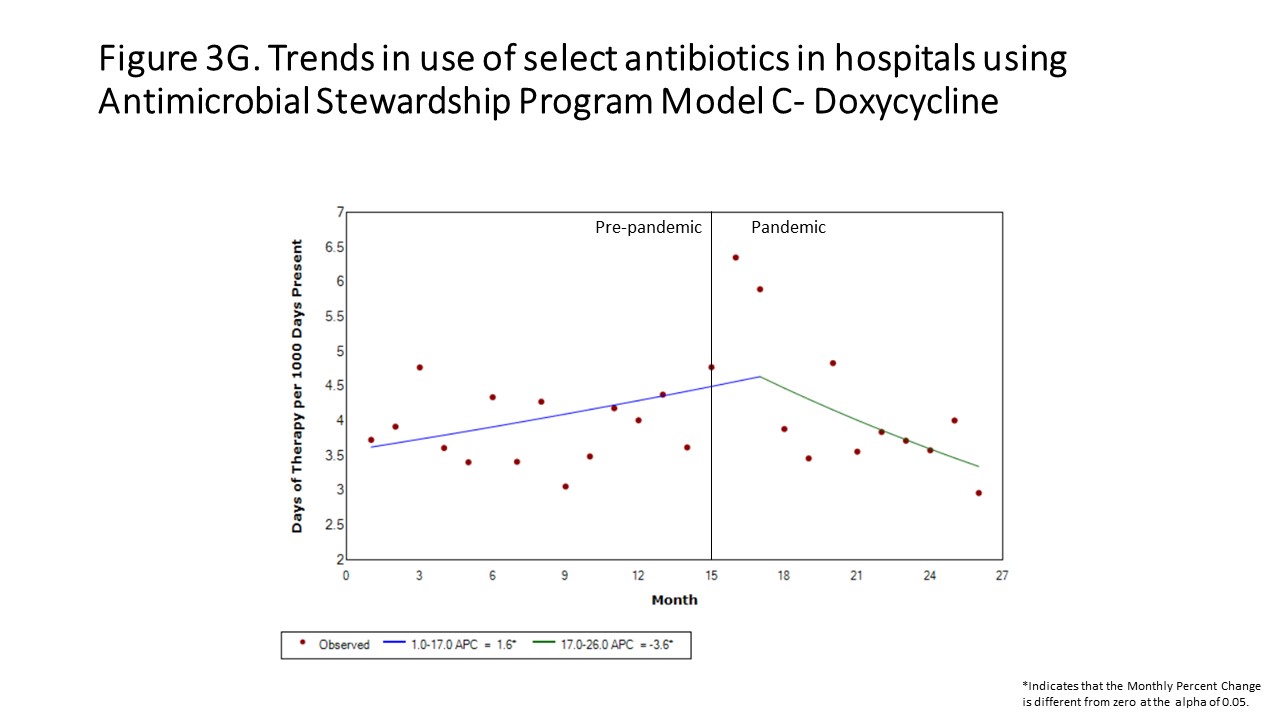


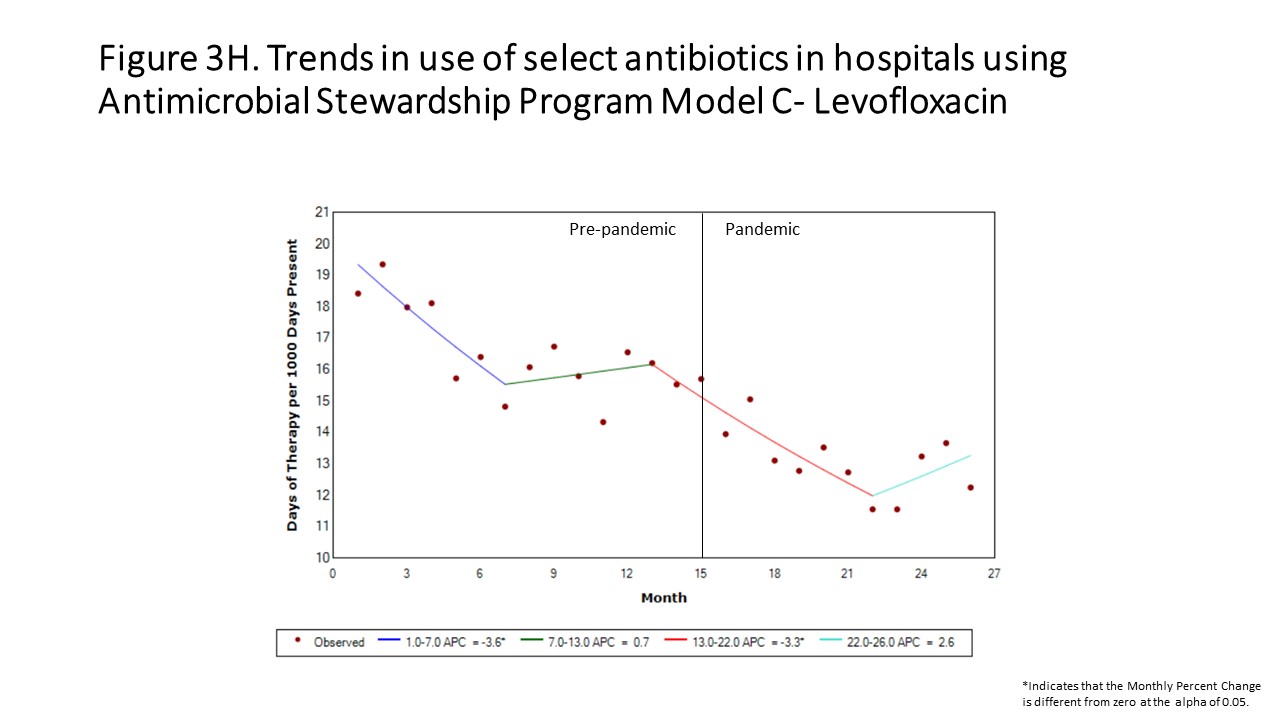

Supplement: Supplementary file 1 [file S2732494X22000390sup001.docx]
